# On Burns and Scalds

**Published:** 1800-04

**Authors:** John Bell

**Affiliations:** Surgeon's Mate. His Majesty's Ship, Castor, Spithead


### *On Burns and Scalds. By Mr. John Bell.*

[ Concluded from our laſt Number, pp. 207. ]

A Caſe that has lately fallen under my obſervation, places the advantages of the ſtimulating plan, in my opinion, upon a moſt ſubſtantial baſis. The infant daughter of a gentleman, who came as a paſſenger in the Caſtor, from Liſbon, (in December laſt) was ſcalded by pulling down a baſon full of hot tea that had been poured out only a moment before. On examination, I found that the whole ſurface of the thorax and abdomen, the upper part of the right thigh, and nearly the whole of the right arm, were ſcalded. In the hurry and confuſion of taking off her cloaths, a portion of the epidermis of the arm had been ſtripped off; the reſt of the ſcalded ſurface was entire, and no bliſter had yet ariſen, for I ſaw her a very few minutes after the accident. The ſcalded parts were copiouſly bathed with *ol. terebinth.* previouſly warmed by putting the phial into hot water, and ſlips of linen ſpread with *ungt. ceræ* afterwards applied; the child was then put to bed. On inquiring at the end of an hour, I was told ſhe was faſt aſleep, and had been ſo for ſome time; at nine o'clock next morning ſhe was ſtill ſleeping, and had reſted remarkably well during the night. Towards evening the plaſters were removed every part was found without bliſters, perfectly covered with the cuticle, (except the right arm) and free from pain, but of a high red colour. The dreſſings of *ungt. ceræ* were again applied, but taken off on the following morning, and never afterwards made uſe of. The arm was duſted with hair powder, which formed a ſcab that fell off in the courſe of a few days, and left the ſkin underneath perfectly ſound.

For an explanation of the principles on which this method of treatment is founded, I muſt refer your readers to Mr. Kentiſh's Eſſay, as it would take up too much of your publication to enter farther on the ſubject.

Mr. Earle's well known profeſſional abilities will doubtleſs operate in favour of his practice; and in caſes where it can be uſed, it will certainly prove in ſome degree beneficial: but I muſt ſay, that I do not think it will ſuperſede the ſtimulating plan, if the merits of each be fairly inveſtigated.

Permit me to thank you for the information I have received from your valuable Journal, on many intereſting ſubjects; it is a work highly uſeful to individuals like myſelf, whoſe ſituation precludes the advantage of an extenſive aſſortment of medical books. I remain,

Gentlemen,

Your obliged humble ſervant,

JOHN BELL, Surgeon's Mate.

